# Enhanced Tolerance to Methyl Viologen-Mediated Oxidative Stress *via AtGR2* Expression From Chloroplast Genome

**DOI:** 10.3389/fpls.2019.01178

**Published:** 2019-09-27

**Authors:** Bipeng Wang, HaiYan Ding, Qiqi Chen, Li Ouyang, Shengchun Li, Jiang Zhang

**Affiliations:** State Key Laboratory of Biocatalysis and Enzyme Engineering, School of Life Sciences, Hubei University, Wuhan, China

**Keywords:** glutathione reductase, reactive oxygen species, chloroplast, methyl viologen, tolerance

## Abstract

Owing to their sessile life habit, plants are continuously subjected to a broad range of environmental stresses. During periods of (a)biotic stresses, reactive oxygen species (ROS) levels can rise excessively, leading to oxidative stress. Glutathione reductase (GR) plays an important role in scavenging the ROS and maintenance of redox potential of the cell during oxidative stress. To enhance ROS scavenging capacity, and hence stress tolerance, the *Arabidopsis thalianaGR2* (*AtGR2*) gene was expressed from the tobacco plastid (chloroplast) genome, the main source of ROS production in plant photosynthetic tissues, in this study. Leaves of transplastomic tobacco plants had about seven times GR activity and 1.5 times total glutathione levels compared to wild type. These transplastomic tobacco plants showed no discernible phenotype and exhibited more tolerance to methyl viologen-induced oxidative stress than wild-type control plants. The results indicate that introducing *AtGR2* in chloroplasts is an efficient approach to increase stress tolerance. This study also provides evidence that increasing antioxidant enzyme *via* plastid genome engineering is an alternative to enhance plant’s tolerance to stressful conditions.

## Introduction

Reactive oxygen species (ROS) are produced as an unavoidable consequence of electron transport processes in photosynthesis and aerobic metabolism. The most common ROS in plants are singlet oxygen (^1^O_2_), hydrogen peroxide (H_2_O_2_), superoxide anion (O_2_^-^), and hydroxyl radical (OH·) (Mittler, 2002). ROS levels rise excessively under stressful conditions, which potentially leads to oxidative stress and damage to macromolecules, such as proteins, lipids, and nucleic acids (Apel and Hirt, 2004). Plants have therefore developed a combination of nonenzymatic and enzymatic antioxidative mechanisms to control ROS concentrations (Mittler, 2002). The ascorbate-glutathione pathway was recognized to be a key player in H_2_O_2_ metabolism in plants, which has been shown to be present in the cytosol, mitochondria, and peroxisomes, as well as the chloroplasts (Foyer and Noctor, 2011; Pandey et al., 2015). In this pathway, the reduced glutathione (GSH) is continuously oxidized to glutathione disulphide (GSSG) that is recycled to GSH *via* NADPH-dependent glutathione reductase (GR) (Noctor and Foyer, 1998). GSH has often been considered to play an important role in defense of plants, in which it functions as a powerful reductant that maintains protein thiols in their reduced state (Noctor et al., 2012; Hasanuzzaman et al., 2017). GR is localized mainly in the chloroplast in leaf tissues, which is also found in the cytosol, mitochondria, and peroxisomes (Noctor et al., 2011). For example, in tobacco and pea plants, about 70% of total GR activity originates from chloroplasts (Edwards et al., 1990; Ding et al., 2009). In *Arabidopsis*, chloroplastic and mitochondrial GR is encoded by *AtGR2*, and *AtGR1* encodes a protein that is localized to both the cytosol and peroxisome (Marty et al., 2009; Mhamdi et al., 2010; Yu et al., 2013).

A plethora of studies have demonstrated that GR plays an essential role in scavenging the ROS and maintenance of redox potential of the plant cell during oxidative stress (Noctor et al., 2012; Gill et al., 2013). It has been observed that GR activity was induced in plants under abiotic and biotic stresses (Kuzniak and Sklodowska, 2001; Jiang and Zhang, 2002; Mittova et al., 2003; Romero-Puertas et al., 2006). Overexpression of GR in plants could effectively increase tolerance to various stresses. Transgenic cotton overexpressing a mutated chloroplastic *AtGR2* resulted in a 36-fold increase in GR activity, coupled with increased tolerance to short-term chilling stress (Kornyeyev et al., 2003; Logan et al., 2003). A recent report has shown that expressing a *GR* from *Stipa purpurea* in *Arabidopsis* resulted in greater tolerance to salt stress than that of wild-type (WT) plants (Wang et al., 2018).

The chloroplast is a major source of ROS because relatively a high concentration of oxygen is able to react with electrons that escape from the photosynthetic electron transfer system (Asada, 2006). Thus, overexpression of antioxidant enzymes in the chloroplast may be a straightforward strategy to increase the tolerance of transgenic plants to various environmental stresses. Tobacco plants expressing the *GR* gene from *Escherichia coli*, targeted to either cytosol or chloroplasts, showed enhanced tolerance to methyl viologen (MV) (Aono et al., 1991, Aono et al., 1993). Similarly, doubled total glutathione levels, signiﬁcant increase in GSH/GSSG ratio, and resistance to MV were observed in the chloroplast-targeted bacterial *GR*–transgenic poplar hybrids (Foyer et al., 1995).

In addition to transform genes encoding ROS scavenging enzymes targeted chloroplasts *via* nuclear transformation, expressing these genes directly in the chloroplast *via* plastid transformation is a promising alternative to protect plants against various oxidative stresses (Le Martret et al., 2011; Poage et al., 2011). Compared with conventional transgenic technologies, plastid transformation offers several highly attractive features, such as site-specific transgene integration, high protein expression levels, the absence of epigenetic and position effects, stacking of transgenes into operons, and the excellent biosafety resulting from maternal inheritance, which greatly prevents the probability of unwanted transgene spread *via* pollen (Bock, 2013; Bock, 2015; Greiner et al., 2015). Thus, plastid engineering has been extensively applied in biopharmaceuticals and industrial enzyme production and in crop improvement against a variety of environmental insults (Chen et al., 2014; Zhang et al., 2015; Adem et al., 2017; Fuentes et al., 2017).

The MV exerts its phytotoxic effects on plants by transferring electrons from photosystem I to O_2_, which leads to the generation of O_2_^-^ in chloroplasts (Kerchev et al., 2015). In this study, we transformed *AtGR2* gene to tobacco chloroplasts genome, resulting in an increase in GR activity and total glutathione pools, as well as enhanced resistance to MV stress.

## Materials and Methods

### Construction of Transformation Vectors

**Figure 1:**
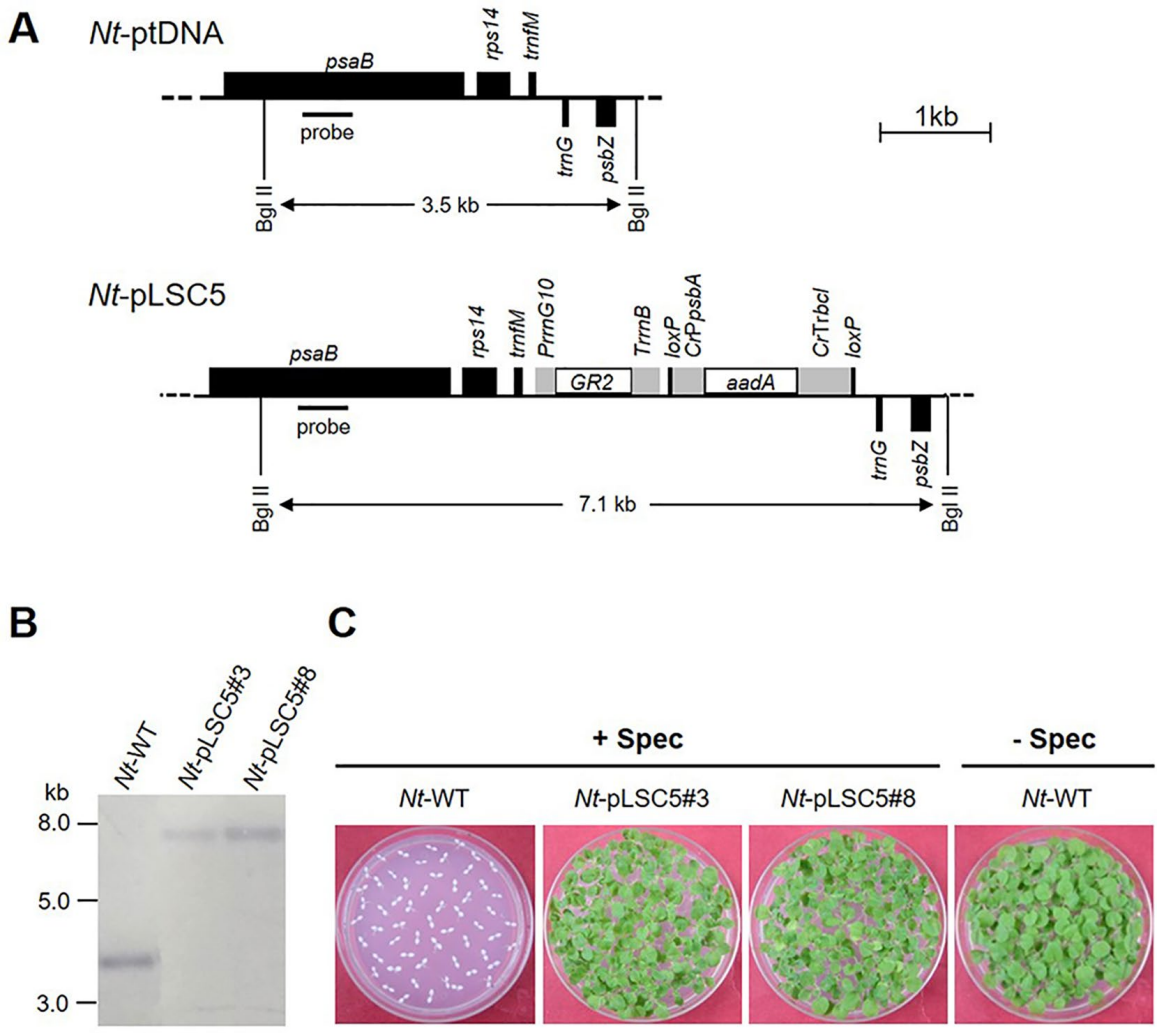
Generation of transplastomic tobacco plants expressing *AtGR2*. **(A)** Physical map of the targeted region in the tobacco plastid genome (*Nt*-ptDNA) and the transgenic plastid genome in *Nt*-pLSC5 lines. The BglII restriction sites used in RFLP analyses and the expected fragment sizes are indicated. The location of the hybridization probe is shown as a black bar. *Cr*, *Chlamydomonas reinhardtii*; *Nt*, *N. tabacum*; T7, bacteriophage T7; P, promoter; T, terminator. **(B)** RFLP analysis of transplastomic *Nt*-pLSC5 plants. The homoplasmic state was evidenced by the absence of the 3.5-kb fragment characteristic of the wild-type (WT) plastid genome and detection of the 7.1-kb fragment specific to the transformed plastid. **(C)** Seed assays confirming the homoplasmic state of the transplastomic plants. Seeds were germinated on medium containing 500 mg/L spectinomycin (*Nt*-wt, *Nt*-pLSC5#3 and *Nt*-pLSC5#8) or antibiotic-free medium (*Nt-*wt).

Total RNA was isolated from 100 mg of Columbia-0 ecotype of *Arabidopsis thaliana* fresh tissue using the Trizol Reagent (Invitrogen, USA). Complementary DNA (cDNA) was synthesized using GoScript™ Reverse Transcription System (Promega, USA) from 3 μg of total RNA. The protocols were performed according to the manufacturer’s instructions. The coding region of the *AtGR2* gene (Locus: AT3G54660), omitting a 222-bp sequence encoding chloroplast transit peptide (Kudo et al., 1993), was amplified by using a primer pair AtGR2-Fwd-1 (TTTAAGAAGGAGATATACCCATGAGTACC GATAATGGAGCTGAATC)/AtGR2-Rev-1 (AGCCTTTCGTTTTATTTGATTCTAG ATTCTACACCCCAGCAGCTGTTTTAG). The primers harbor (underlined) a short homologous sequence to a plastid expression vector pYY12 (Wu et al., 2017). The pYY12 was digested using NcoI and XbaI (Takara, Japan) and then cotransformed with *AtGR2* PCR product into *E. coli* (XL10-Gold, Agilent technologies) competent cells to generate plastid transformation construct pLSC5 (Wu et al., 2017) (**Figure 1A**).

### Plant Material and Growth Conditions

Tobacco seedlings (*Nicotiana tabacum* L. cv Petit Havana) were raised on agar-solidified MS medium containing 30 g/L sucrose (Murashige and Skoog, 1962). Regenerated homoplasmic shoots from transplastomic *N. tabacum* lines were rooted and propagated on the same medium. Rooted plants were transferred to soil and cultivated in a controlled environment growth room (light intensity: 150 μmol m^−2^ s^−1^; day temperature: 25^◦^C; night temperature: 20 ^◦^C; diurnal cycle: 16-h light/8-h darkness; relative humidity: 55%).

### Plastid Transformation and Selection of Transplastomic Lines

DNA for plastid transformation was prepared using the Nucleobond Xtra Plasmid Midi Kit (Macherey-Nagel, Germany). Young leaves from aseptically grown tobacco plants were bombarded with plastid-coated 0.6-µm gold particles (BioRad) using a helium-driven biolistic gun (PDS-1000 He; Bio-Rad) with the Hepta Adaptor setup and 1,100 psi rupture disks (BioRad). The bombarded leaf samples were cut into small (∼5 × 5 mm) pieces and were placed with abaxial side up on RMOP medium containing 500 mg/L spectinomycin in a Petri dish for selection (Svab and Maliga, 1993). In general, spectinomycin-resistant shoots appeared within 1 month. The initial shoots were further screened on RMOP medium supplemented with spectinomycin (500 mg/L) and streptomycin (500 mg/L) to eliminate spontaneous spectinomycin-resistant mutants. Several independent transplastomic lines were obtained and subjected to one or two additional rounds of regeneration on RMOP/spectinomycin medium to select for homoplasmy. Homoplasmic shoots were transferred onto rooting medium, which consists of MS salts, 30 g/L sucrose, and 0.1 mg/L NAA. Rooted shoots were transferred to the greenhouse for seed production.

### DNA Extraction and Southern Blot Analysis

Total DNA was isolated from leaves of WT and spectinomycin-resistant plants using a cetyltrimethyl ammonium bromide (CTAB)-based extraction method (Clarke, 2009). DNA samples (5 µg total cellular DNA) were digested with Bgl II for 16 h, separated by electrophoresis in 1% agarose gels and transferred onto a positively charged nylon membranes (GE Healthcare, USA) by capillary action using the semi-dry transfer method. A 550-bp fragment of the *psaB* gene was amplified by PCR from tobacco plastid DNA using primer pair psaB-Fwd (CCCAGAAAGAGGCTGGCCC)/psaB-Rev (CCCAAGGGGCGGGAACTGC) and used as hybridization probe to verify plastid transformants. Labeling of the probe and hybridization were performed with the DIG-High Prime DNA Labeling and Detection Starter Kit II following the manufacturer’s instructions (Roche, USA).

### RNA Isolation and Northern Blot Analysis

Total RNA was isolated from fresh leaves of WT and transplastomic plants using Trizol Up (TransGen Biotech, China) and following the manufacturer’s instructions. RNA samples (3 µg total RNA) were denatured and separated by electrophoresis in formaldehyde-containing 1% agarose gels. The separated RNA molecules were then transferred from the gel to a positively charged nylon membrane (GE Healthcare, USA). The *AtGR2* specific probe, a 566-bp fragment, was ampliﬁed by PCR from cDNA of *Arabidopsis* with primer pair AtGR2-Fwd-2 (CCTGGTTGGAGCCCTCATAGT)/AtGR2-Rev-2 (GCAAGCCCAACACAAAGAACT). The probe was labeled with DIG using the PCR DIG probe synthesis kit following the manufacturer’s protocol (Roche, USA). RNA blots were hybridized at 42^◦^C.

### Methyl Viologen Treatment

Leaf discs (10 mm in diameter) were cut out from leaves of nodes 3 and 4 of soil-grown plants and floated, with abaxial surface down, on 10 ml of water or solutions containing different concentrations of MV (0.5, 1, 2, and 5 μM) in 24-well plate. Plates were incubated in the dark for 12 h to allow diffusion of the MV into the leaf disc and then placed under light (intensity 100 μmol m^−2^ s^−1^) for 24 h.

### Ion Leakage Measurement

The ion leakage into the solutions used for ﬂoating the leaf discs in the MV experiment was determined using the Leici DDS-11A conductivity meter (Shanghai, China). The conductivity was measured ﬁrst after each experiment (value A), then the bathing solutions containing the leaf discs were autoclaved at 121°C for 20 min, and the conductivity was measured again to get the total ion leakage (value B). The relative ion leakage was then calculated as a percentage: (value A⁄value B) × 100.

### Estimation of Chlorophyll Content

The concentrations of chlorophyll a and chlorophyll b were calculated based on the formula of Kirk and Allen (1965).

$$\begin{array}{l} \text{Chlorophyll a}=12.7\times {\text{A}}_{663}–2.49\times {\text{A}}_{645}\\ \text{Chlorophyll b}=22.9\times {\text{A}}_{645}–4.68\times {\text{A}}_{663} \end{array}$$

### Antioxidant Enzyme Assays, Metabolites, and ROS Analysis

Extractable enzyme activities were measured as described previously (Noctor et al., 2016). Oxidized and reduced forms of glutathione were measured by plate-reader assay as described by Le Martret et al. (2011). H_2_O_2_ content was determined by the method of titanium oxidation with hydroperoxide-titanium complex formed (Brennan and Frenkel, 1977).

## Results

### Generation of Transplastomic Tobacco Lines

The plastid transformation vector pLSC5 (**Figure 1A**) was constructed, and *AtGR2* was introduced into tobacco plastids *via* particle bombardment, and transplastomic shoots were regenerated on a synthetic regeneration medium containing spectinomycin. A number of transplastomic lines were obtained and subjected to one or two additional regeneration rounds on spectinomycin-containing RMOP medium. Two independently transformed lines (*Nt*-pLSC5#3 and *Nt*-pLSC5#8) were further characterized. Southern blot analysis veriﬁed the homoplasmic state of the *Nt*-pLSC5 lines, as evidenced by absence of a hybridization signal for the 3.5-kb fragment diagnostic of the WT and exclusive presence of the 7.1-kb fragment expected in transplastomic plants (**Figure 1B**). The homoplasmic state of the transplastomic lines was further verified by seed assays (**Figure 1C**).

The phenotypes of homoplasmic transplastomic lines were analyzed under both mixotrophic growth conditions on synthetic sucrose-containing medium (**Figure S1A**) and autotrophic growth conditions in soil (**Figure S1B**). Transplastomic *Nt*-pLSC5 plants were indistinguishable from WT plants in both conditions. Measurement of chlorophyll from autotrophic grown plants additionally confirmed the indistinguishable phenotype between *Nt*-pLSC5 and WT plants (**Figure S1C**).

### Glutathione Reductase and Glutathione Analysis

**Figure 2:**
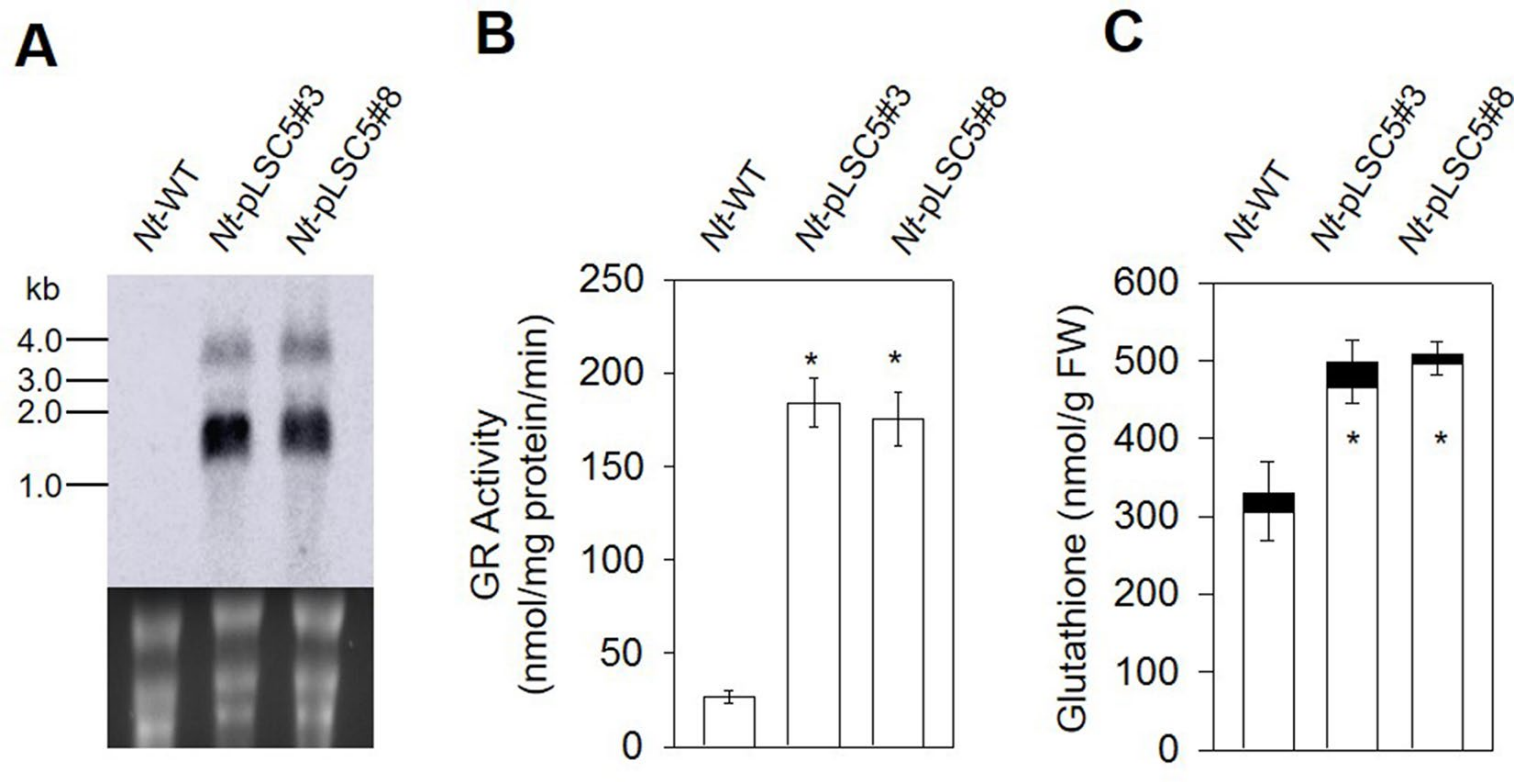
Glutathione reductase and glutathione analysis in the leaves of wild-type (WT) and *Nt*-pLSC5 plants. **(A)** Northern blot to analyze mRNA accumulation in transplastomic lines expressing *AtGR2*. Below each blot, the rRNA-containing region of the ethidium bromide-stained gel prior to blotting is shown as a control for RNA integrity and equal loading. Larger bands represent unprocessed polycistronic precursor transcripts and read-through transcripts (which are common in plastids; e.g., Elghabi et al., 2011; Zhou et al., 2008). **(B)** GR activity and **(C)** of glutathione content of WT and transplastomic lines. White bars, reduced forms. Black bars, oxidized forms. Data are means ± SE of four independent extracts. Asterisks indicate signiﬁcant differences from WT at P < 0.05.

To examine the *AtGR2* transcript accumulation in leaves of *Nt*-pLSC5 plants, Northern blotting was performed using a hybridization probe specific for the *AtGR2* coding region. Hybridization to the *AtGR2* specific probe detected two transcripts (**Figure 2A**), with the smallest of ∼1.5 kb representing the expected *AtGR2* mRNA. The larger bands most probably represent read-through transcripts, which are common in plastids and usually originate from ineﬃcient transcription termination (Zhou et al., 2008; Elghabi et al., 2011).

In leaves of the *Nt*-pLSC5 plants, the GR activity increased six-fold compared to WT (**Figure 2B**). To further assess the impact of enhanced GR activity on the ROS–antioxidant interaction, we measured the extractable activities of ascorbate peroxidase (APX), dehydroascorbate reductase (DHAR), catalase (CAT), and superoxidase dismutase (SOD), the enzymes linking H_2_O_2_ metabolism and glutathione pools (Noctor and Foyer, 1998). CAT activity was significantly increased in *Nt*-pLSC5 plants compared to WT (**Figure S2A**), while APX, DHAR, and SOD activities were not greatly affected (**Figures S2B, C, D**).

The level of total glutathione was elevated by ∼50% in *Nt*-pLSC5 plants when compared to WT. This increase was mostly due to an increase in the levels of GSH (**Figure 2C**).

### Effect of Methyl Viologen Stress

**Figure 3:**
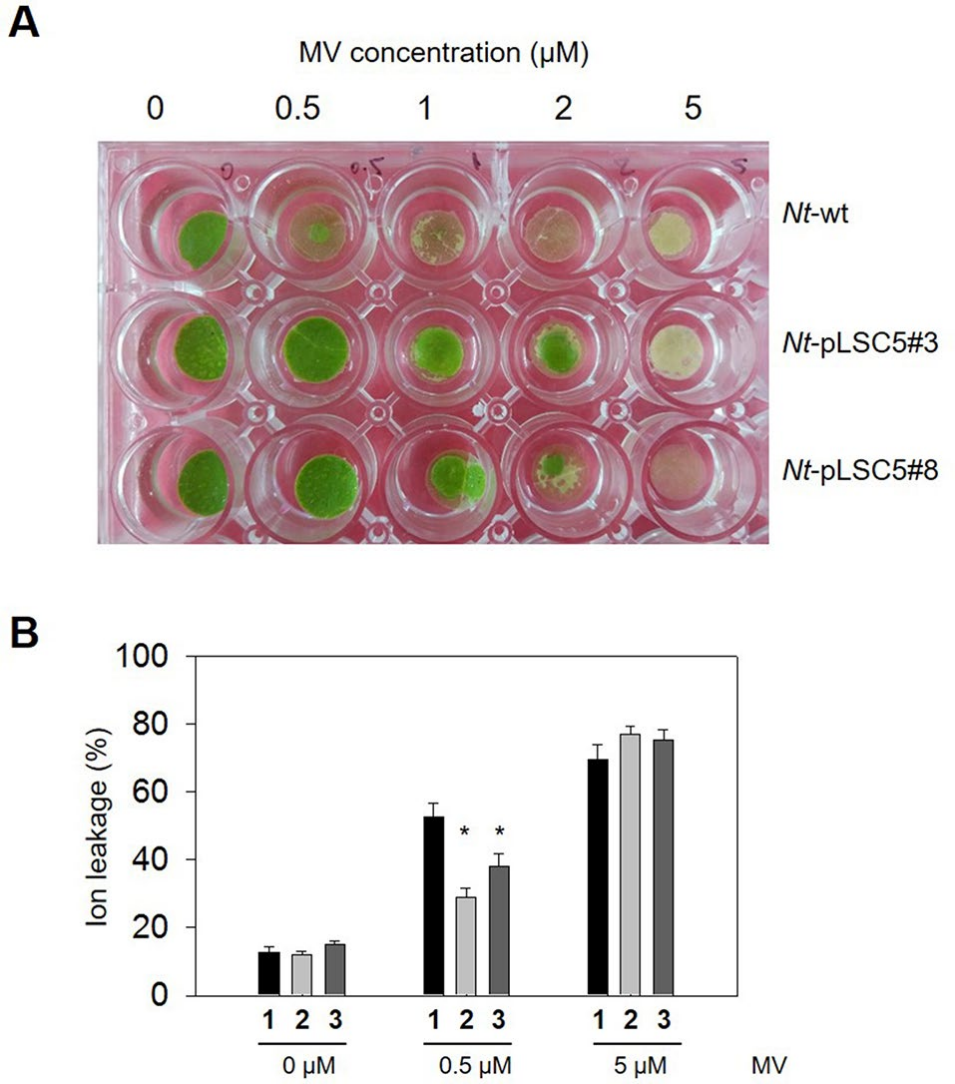
Effect of methyl viologen(MV) on leaf discs of wild-type (WT) and transplastomic tobacco plants. **(A)** Representative image showing phenotypic differences of WT and *Nt*-pLSC5 transgenic plant leaf discs ﬂoated on a 0.5-, 1-, 2-, and 5-µM MV solution after 24 h illumination. **(B)** Analysis of membrane damage measured by ion leakage. The experiments were repeated four times, and data are means ± SE of four independent measurements. Asterisks indicate signiﬁcant differences from WT at P < 0.05. **1**, *Nt*-wt; **2**, *Nt*-pLSC5#3; **3**, *Nt*-pLSC5#8.

To evaluate tolerance to MV-mediated oxidative stress, transplastomic, and WT leaf discs were ﬂoated on MV solution with concentrations. Severe necrosis was observed in WT leaf discs treated with MV from 0.5 μM upward, while partial necrosis was observed and increased in a dose-dependent manner at the edges of transplastomic leaf discs up to 2 μM MV. All leaf discs were strongly damaged at 5 μM MV (**Figure 3A**). The membrane damage in MV-treated, WT, and transplastomic leaf discs was measured by ion leakage. A protective effect was detected in the transplastomic leaf discs exposed to 0.5 μM MV, which showed a significantly lower percentage of ion leakage compared to WT, and no signiﬁcant difference in the percent of leakage was seen between WT and transplastomic leaf discs treated with 5 μM MV (**Figure 3B**).

**Figure 4:**
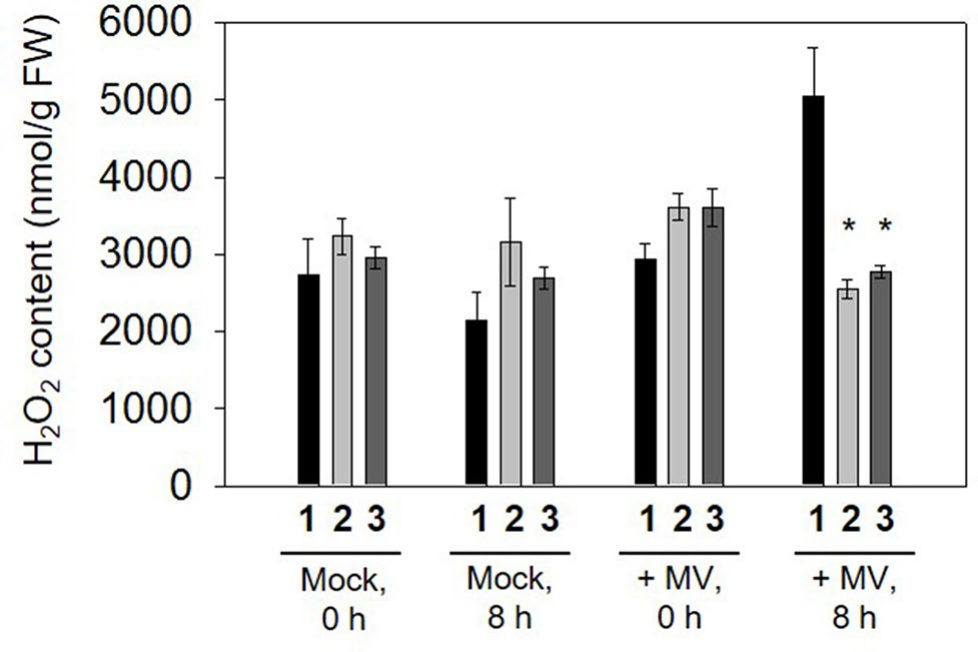
H_2_O_2_ content measured in leaf discs incubated in 2 µM methyl viologen (MV) following 0 and 8 h illumination. Data are means ± SE of four independent extracts. Asterisks indicate signiﬁcant differences from wild type (WT) at P < 0.05. **1**, *Nt*-wt; **2**, *Nt*-pLSC5#3; **3**, *Nt*-pLSC5#8.

To determine whether overexpressing GR had any inﬂuence on H_2_O_2_ accumulation during MV stress exposure, the amount of H_2_O_2_ was also measured in control and transplastomic leaf discs in the presence or absence of stress. The H_2_O_2_ accumulated markedly in WT following 8 h illumination of leaf discs incubated with 2 μM MV, while H_2_O_2_ content remained relatively lower in transplastomic leaf discs (**Figure 4**), suggesting that enhanced GR activity in transplastomic plants contributes to control excessive H_2_O_2_ levels.

## Discussion

As a component of ascorbate-glutathione pathway, GR plays an essential role in ROS detoxiﬁcation, GSH regeneration, and stress tolerance in plants (Gill et al., 2013). Overexpressing the *GR* gene led to an increase in antioxidant capacity and, hence, stress tolerance. The chloroplast is regarded as an important source of ROS production due to numerous electron transfer reactions in the presence of oxygen, which would be further accelerated by stresses (Foyer and Noctor, 2003; Waszczak et al., 2018). Overexpression of *E. coli GR* in chloroplasts was more effective than that in cytosol under abiotic stresses. For example, overexpression of bacterial *GR* in the chloroplast of poplar resulted in 1,000 times higher GR activity; however, cytosolic *GR*-transformed poplar had only 10 times higher GR activity compared to control plants. Moreover, only chloroplastic *GR*-transformed plants exhibited an increase in GSH/GSSG ratio and glutathione levels and more tolerance to photoinhibition as well (Foyer et al., 1995). Likewise, Indian mustard overexpressing bacterial *GR* gene in the chloroplast, but not in the cytosol, accumulated twice higher glutathione levels in roots and showed enhanced cadmium tolerance (Pilon-Smits et al., 2000). The higher effectiveness of chloroplast-targeted than cytosol-targeted bacterial *GR* overexpression in plants was attributed to higher stability of the *E. coli* protein in the chloroplast than in the cytosol (Foyer et al., 1995).

In this study, we report the stable integration and expression of *AtGR2* gene from the tobacco plastid genome. The transplastomic *Nt*-pLSC5 plants had an increase in both GR activity and GSH level (**Figures 2B, C**), resulting in more tolerance to MV treatment compared to WT (**Figure 3**). Increased activity of CAT could make a contribution to MV tolerance through H_2_O_2_ degradation (**Figure S2B**). However, these results conflicted with a previous report that more oxidative stress sensitivity to MV treatment was found in *E. coli* GR–transplastomic tobacco plants than untransgenic control (Poage et al., 2011). Although the *AtGR2* and bacterial *GR*–transplastomic lines had similar GR activity increase, total glutathione pools were much higher (eight-fold increase) in the latter ones (Poage et al., 2011). In fact, eight-fold total glutathione levels increase was the most powerful induction in *GR*-transformed plants in available literatures (Mullineaux and Rausch, 2005). The more MV sensitivity of bacterial *GR*–transplastomic was explained on the basis of impaired redox sensing in the chloroplast, which was similar to the finding of Creissen et al. (1999) who increased three-fold GSH level by transformation with γ-glutamylcysteine synthetase (γ-ECS) targeted to chloroplasts (Creissen et al., 1999). These results suggest that there may be a threshold value of glutathione concentration in chloroplasts in plants beyond which would lead to oxidative stress on the contrary. Intriguingly, overexpressing bacterial *GR* in the chloroplast of poplar led to about twice increase in GSH level, despite that extractable GR activity was up to 1,000-fold higher than those of control poplars (Foyer et al., 1995), which suggests that GSH correlates weakly with GR activity. After all, glutathione pools are also affected by γ-ECS activity and cysteine availability (Noctor et al., 2012).

Moreover, plant-encoded GR affords greater protection than the *E. coli*-derived enzyme may partially explain the contrasting observation between *AtGR2*- and bacterial *GR*–transplastomic tobacco plants (Broadbent et al., 1995). Overexpression of a pea *GR* gene in the tobacco chloroplast, cytosol, or in both chloroplasts and mitochondrion simultaneously resulted in elevated GR activity and a 50% increase in total glutathione pools, which tolerated much higher MV concentration than the bacterial *GR*-transgenic tobacco plants (Aono et al., 1991, Aono et al., 1993), suggesting that plant-encoded *GR* might afford greater protection than the *E. coli*-derived enzyme. This may be due to the fact that GRs from plant sources is less sensitive to inhibition by NADPH than *E. coli*-derived GR (Mata et al., 1984; Connell and Mullet, 1986; Mahan and Burke, 1987).

Besides tolerance to MV, which induces excessive ROS generation in chloroplasts, transplatomic *Nt*-pLSC5 plants also showed enhanced tolerance to osmotic stress (**Figure S3**). Elevated GR was reported to improve heavy metal stress due to the observed upgraded GSH levels, which is the precursor for the metal binding peptides, phytochelatins (Pilon-Smits et al., 2000; Poage et al., 2011). However, transplatomic *Nt*-pLSC5 plants did not exhibit tolerance to cadmium and lead compared to WT plants probably due to insufficient induction of the glutathione pool (**Figure S4**).

Apart from different sources of GR selection and its expression in different cellular compartments, recognizing characteristics of GR isoforms, including their monomeric, homodimeric, and heterodimeric structures, is also crucial for GR gene manipulation in plants under stress conditions (Yannarelli et al., 2007; Gill et al., 2013). Moreover, increasing evidences indicated that transgenic plants with multiple genes for antioxidant pathway showed better tolerance to environmental stresses compared to that containing a single gene product. For example, simultaneous overexpression of *GR* and *SOD* in tobacco exhibited much more tolerance to MV than that transformed single *GR* (Aono et al., 1995). Coexpression of *GR* and *DHAR* or glutathione-*S*-transferase from tobacco plastid genome resulted in the increased tolerance of the transgenic plants to a variety of abiotic stresses, such as MV-induced oxidative stress, salt, cold, and heavy metal stresses, whereas expression of sole *GR* exhibited sensitivity to MV (Le Martret et al., 2011). As multiple genes can be engineered as an operon in plastid genome, therefore, next effort would be combined. In conclusion, our study clearly indicates that directly expressing ROS-scavenging enzyme in plastid is an attractive approach to increase plant’s tolerance to stress conditions.

## Author Contributions

SL and JZ conceived and designed the research. BW, HD, QC, and LO conducted the experiments. SL and JZ analyzed the data and wrote the manuscript. All authors read and approved the manuscript.

## Conflict of Interest

The authors declare that the research was conducted in the absence of any commercial or financial relationships that could be construed as a potential conflict of interest.

## Supplementary Material

Figure S1 Phenotypes of WT and transplastomic plants. Plants are shown after **(A)** 6 weeks of growth under heterotrophic conditions in sterile culture on sucrose-containing synthetic medium, **(B)** 6 weeks of growth under photoautotrophic conditions in soil. Bars indicate 2 cm. **(C)** Chlorophyll a/b contents of leaves from **(C)** cultivated condition. Data are means ± SE of four independent extracts.

Figure S2 Activities of antioxidative enzymes related to H_2_O_2_ metabolism and glutathione pools in WT and transgenic plants. **(A)** APX. **(B)** CAT. **(C)** DHAR. **(D)** SOD. The experiments were repeated four times and data are means ± SE of four independent measurements. Asterisks indicate significant differences from WT values in the same condition (P < 0.05).

Figure S3 Osmotic tolerance analysis of *AtGR2*-transplatomic tobacco plants. Representative pictures of WT and transplastomic *Nt*-pLSC5 plants seeds germinated on 1/2 MS medium containing 0 or 300 mM mannitol for 10 days, 17 days and 24 days **(A)**, and the germination rate was calculated **(B)**. Vertical bars indicate means ± SE calculated from four independent biological replicates with similar results. Asterisks indicate signiﬁcant differences from WT at P< 0.05. **1**, *Nt*-wt; **2**, *Nt*-pLSC5#3.

Figure S4 Heavy metal tolerance test of AtGR2-transplatomic tobacco plants. The WT and the transplastomic *Nt*-pLSC5 plants seeds were germinated on 1/2 MS medium containing 0 **(A)**, 0.2 mM **(B)** or 0.3 mM **(C)** CdCl_2_, 1.0 mM **(D)** or 1.5 mM **(E)** PbAc_2_ for three weeks. The experiments were repeated three times.
