# Supplementary figures and images for: Enhanced Tolerance to Methyl Viologen-Mediated Oxidative Stress via AtGR2 Expression From Chloroplast Genome
Source: Front Plant Sci. 2019 Sep 27;10:1178. doi: 10.3389/fpls.2019.01178 (PMC6777472; doi:10.3389/fpls.2019.01178)

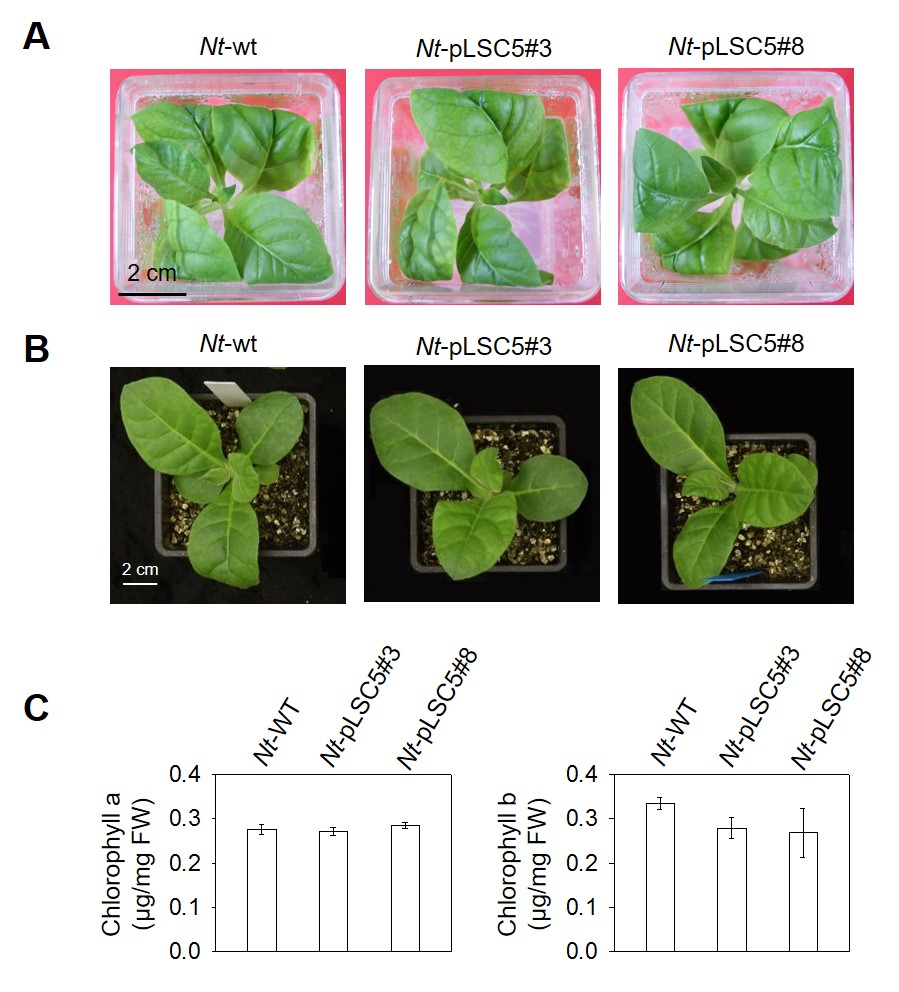

Supplement: Figure S1 — Phenotypes of WT and transplastomic plants. Plants are shown after (A) 6 weeks of growth under heterotrophic conditions in sterile culture on sucrose-containing synthetic medium, (B) 6 weeks of growth under photoautotrophic conditions in soil. Bars indicate 2 cm. (C) Chlorophyll a/b contents of leaves from (C) cultivated condition. Data are means ± SE of four independent extracts. [file Image_1.jpeg]

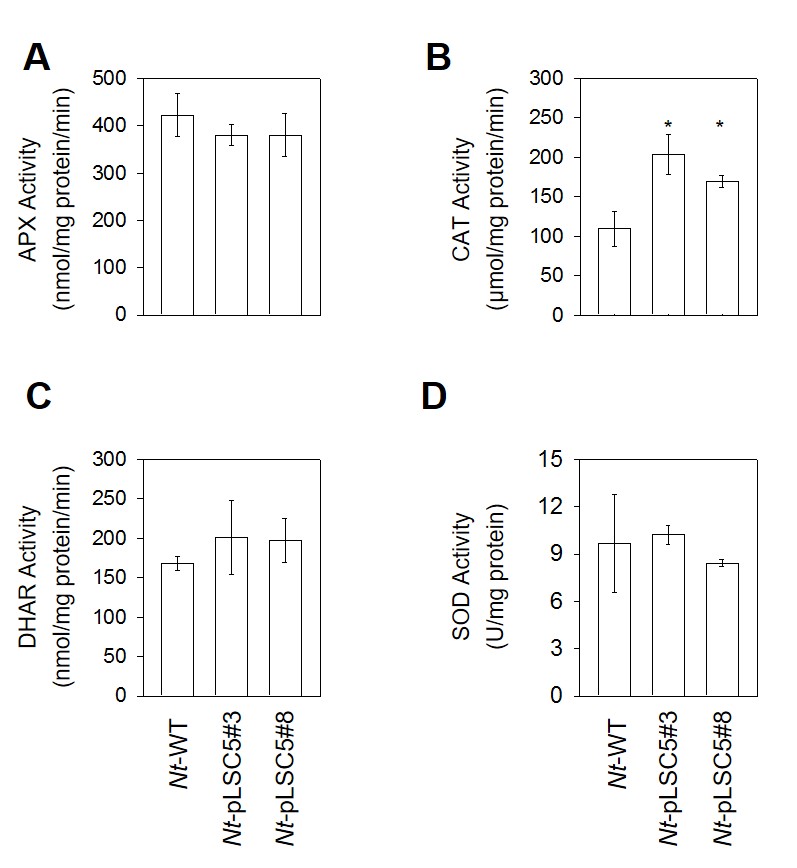

Supplement: Figure S2 — Activities of antioxidative enzymes related to H2O2 metabolism and glutathione pools in WT and transgenic plants. (A) APX. (B) CAT. (C) DHAR. (D) SOD. The experiments were repeated four times and data are means ± SE of four independent measurements. Asterisks indicate significant differences from WT values in the same condition (P < 0.05). [file Image_2.jpeg]

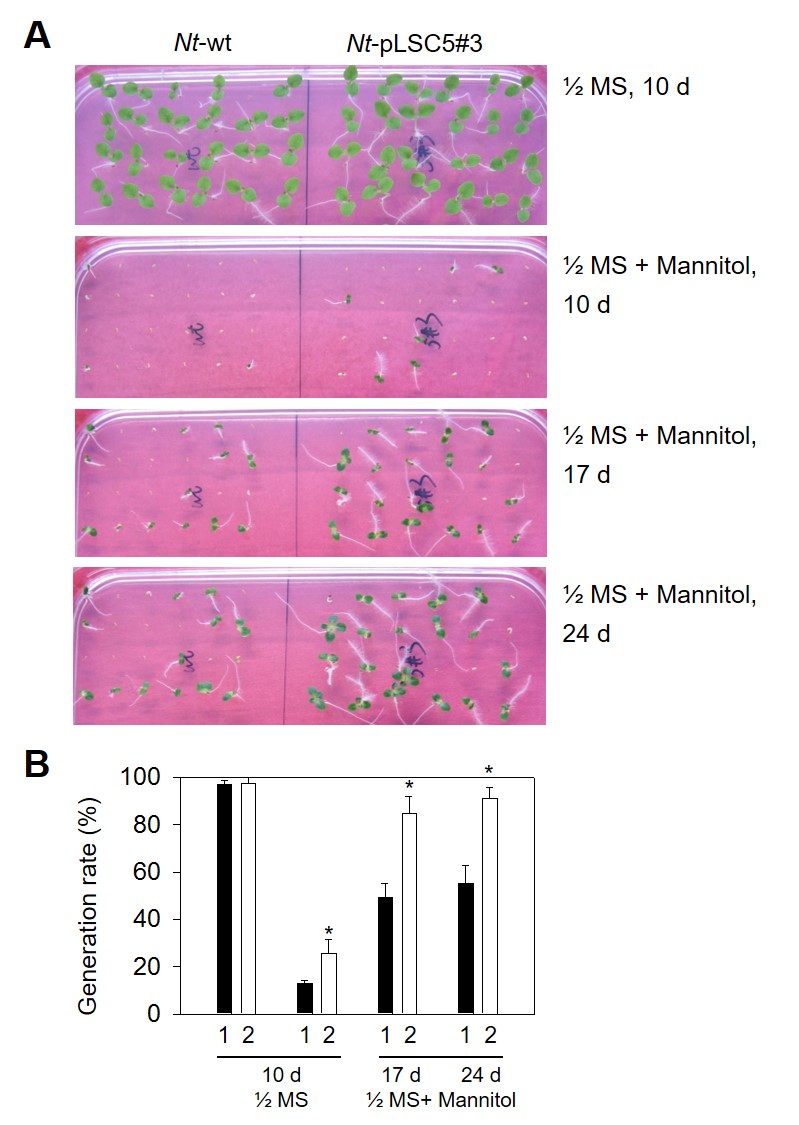

Supplement: Figure S3 — Osmotic tolerance analysis of AtGR2-transplatomic tobacco plants. Representative pictures of WT and transplastomic Nt-pLSC5 plants seeds germinated on 1/2 MS medium containing 0 or 300 mM mannitol for 10 days, 17 days and 24 days (A), and the germination rate was calculated (B). Vertical bars indicate means ± SE calculated from four independent biological replicates with similar results. Asterisks indicate signiﬁcant differences from WT at P< 0.05. 1, Nt-wt; 2, Nt-pLSC5#3. [file Image_3.jpeg]

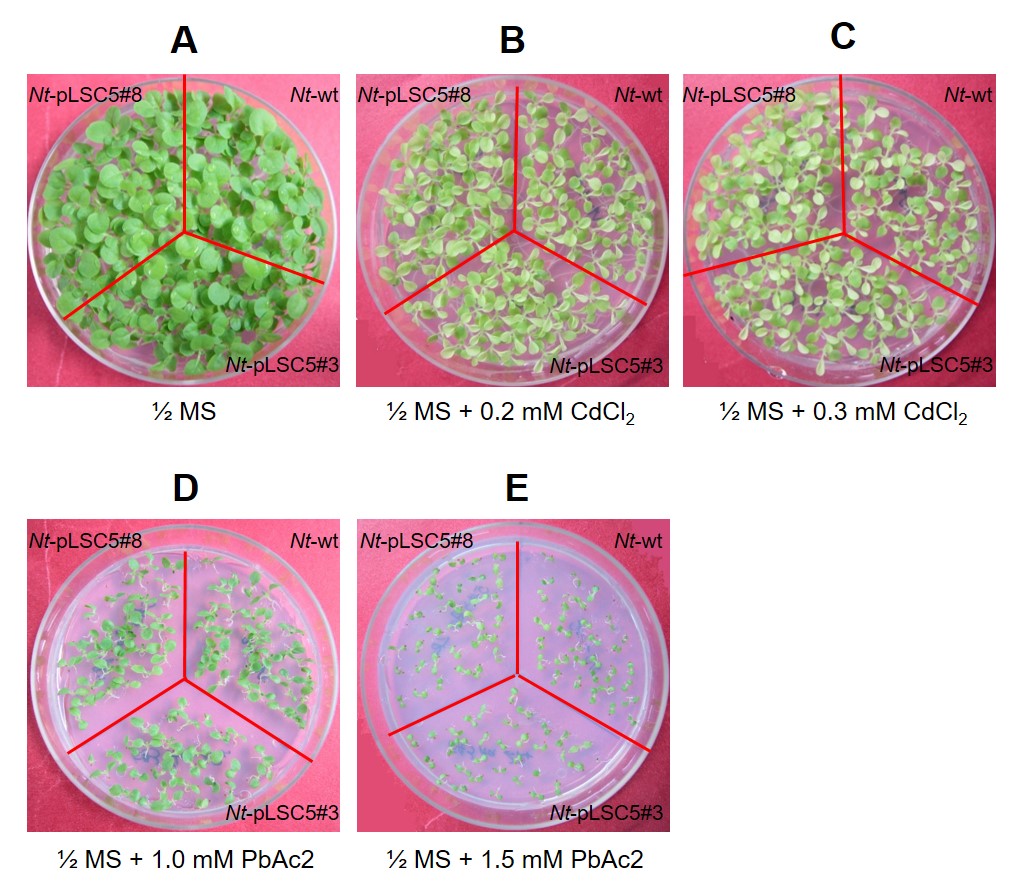

Supplement: Figure S4 — Heavy metal tolerance test of AtGR2-transplatomic tobacco plants. The WT and the transplastomic Nt-pLSC5 plants seeds were germinated on 1/2 MS medium containing 0 (A), 0.2 mM (B) or 0.3 mM (C) CdCl2, 1.0 mM (D) or 1.5 mM (E) PbAc2 for three weeks. The experiments were repeated three times. [file Image_4.jpeg]
